# Impact of different sulfur sources on the structure and function of sulfur autotrophic denitrification bacteria

**DOI:** 10.1038/s41598-023-46829-y

**Published:** 2023-11-08

**Authors:** Zhenguo Chen, Minlan Lou, Peizhen Fang, Dunquan Xiao, Wenting Zhu, Hongwei Chen, Wei Qian

**Affiliations:** 1 School of Chemistry and Life Sciences, Suzhou University of Science and Technology, Suzhou, China; 2 Suzhou Fangzhou Environmental Protection Technology Co., Ltd, Suzhou, China; 3 Zhejiang Construction Environmental Protection Engineering Co., Ltd, Hangzhou, China; 4 State Key Laboratory of Pollution Control and Resource Reuse, School of the Environment, Suzhou Polytechnic Institute of Agriculture, Suzhou, China

**Keywords:** Water microbiology, Environmental biotechnology

## Abstract

Nitrate pollution in surface water has become a significant environmental concern. Sulfur autotrophic denitrification (SAD) technology is gaining attention for its cost-effectiveness and efficiency in nitrate removal. This study aimed to investigate the structure and function of sulfur autotrophic denitrification microbial communities in systems using sodium thiosulfate (Group A) and elemental sulfur (Group B) as the sole electron donors. Metagenomic amplicon sequencing and physicochemical analysis were performed to examine the microbial communities. The results revealed that on day 13, the nitrate nitrogen removal rate in Group A was significantly higher (89.2%) compared to Group B (74.4%). The dominant genus in both Groups was *Thiobacillus*, with average abundances of 34.15% and 16.34% in Groups A and B, respectively. β-diversity analysis based on species level showed significant differences in bacterial community structure between the two Groups (P < 0.001). Group A exhibited a greater potential for nitrate reduction and utilized both thiosulfate and elemental sulfur (P < 0.01) compared to Group B. This study provides a sufficient experimental basis for improving the start-up time and operating cost of SAD system through sulfur source switching and offers new prospects for in-depth mechanistic analysis.

## Introduction

The rapid growth of the economy and development has led to an escalating issue of uncontrolled or substandard discharge of industrial, agricultural, and domestic sewage^1, 2^. This discharge introduces nutrients such as nitrogen and phosphorus into the surface and underground water bodies. These substances are carried into rivers and lakes through surface runoff, serving as the primary cause of eutrophication in these water bodies and others^3^.

In water bodies, nitrogen exists in different forms and undergoes natural transformations. Ultimately, most forms of nitrogen tend to stabilize as nitrate nitrogen. As a result, nitrate nitrogen compounds are the predominant nitrogen compounds in natural water bodies. In slightly polluted water bodies, nitrate nitrogen content can account for more than 60% of the total nitrogen present. The excessive presence of nitrate nitrogen poses a significant risk to human health, agriculture, and fisheries, making nitrate nitrogen pollution a pressing environmental pollution that requires attention and resolution^4^.

Nitrate pollution has been addressed using biological methods due to their cost-effectiveness and removal efficiency. These methods rely on the denitrification abilities of microorganisms, which convert nitrate nitrogen into nitrogen and release it into the atmosphere. The primary challenge in treating nitrate-contaminated wastewater using microbial processes lies in the low C/N ratio. Heterotrophic microorganisms require organic carbon sources as well as nitrate as electron acceptors and donors to facilitate the biological reduction process that converts nitrate to nitrogen^5^. Adding excessive organic carbon sources can lead to secondary pollution due to residual organic matter in wastewater.

Conversely, autotrophic denitrification is a process where autotrophic microorganisms utilize inorganic carbon compounds (e.g., CO_2_ and HCO_3_^-^) and inorganic substances such as Fe(II), hydrogen, and reduced sulfides as the carbon source and electron donor for denitrification, respectively^6–8^. The growth rate of most autotrophic denitrification bacteria is slower due to their requirement for an anaerobic environment. However, they offer distinct advantages over other pathways, such as the absence of secondary pollution and lower sludge production. Utilizing reduced sulfides as the electron donor among various substrates provides additional benefits, including a simplified process, ease of adjustment, and enhanced safety measures. Sulfur autotrophic denitrification shows great potential in treating wastewater with a low C/N ratio. This includes various types of wastewater, such as primary sewage effluent, groundwater, landfill leachate, etc.

The primary factor impeding the widespread adoption of sulfur autotrophic denitrification technology in practical engineering applications is the extended start-up time of the reactor due to the slow growth of sulfur autotrophic denitrification bacteria and low availability toward microorganisms^9, 10^. Previous studies only compared the denitrification efficiency and species composition (genus level) of denitrification systems with different sulfur sources. To address these issues, we have developed a cultivation plan for sulfur autotrophic microorganisms utilizing different substrates, namely thiosulfate and elemental sulfur and investigated from the perspective of species level and functional genes. Our specific objectives for this study are as follows: (1) to examine the temporal changes in denitrification efficiency within the reactors, (2) to elucidate the disparities and connections between the two microbial communities at the species level, and (3) to evaluate the variations in sulfur and nitrogen metabolism functions between the two communities and assess the feasibility of their combined application in practical engineering.

## Results

### Temporal changes in physical and chemical profiles during domestication

**Figure 1:**
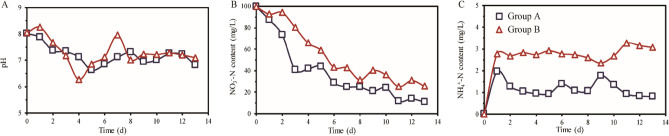
A study of the dynamics of physiochemical properties includes pH (**A**), NO_3_^-^-N content (**B**) and NH^4^^+^-N content (**C**) in sulfur autotrophic denitrification bacteria domestication. The square and triangle represent samples from Groups A and B, respectively.

Homologous changing patterns of physicochemical properties during the domestication process were observed in both Group A and Group B (Fig. 1). Initially, the pH levels of both Groups decreased to 6.63 and 6.26, respectively, as sulfur autotrophic denitrification consumes alkalinity. Subsequently, the pH gradually rose and maintained a small fluctuation, reaching around 7.1 (Fig. 1A). When the hydraulic retention time was set to 2 days for both Groups, the effluent’s nitrate nitrogen content rapidly decreased. Notably, the nitrate nitrogen removal rate of Group A was significantly higher than Group B (P < 0.05) (Fig. 1B). On the 13th day, the nitrate nitrogen removal rates in the two Groups were 89.2% and 74.4%, respectively. Some nitrate nitrogen underwent conversion into ammonia nitrogen through assimilatory nitrate reduction in the anaerobic systems. In our experiment, ammonia nitrogen was detected in the system after 1 days of cultivation, but the concentration remained relatively low. The average concentration of ammonia nitrogen in the two groups was 1.11 mg/l and 2.81 mg/l, respectively (Fig. 1C). Based on the changes in nitrate nitrogen and ammonia nitrogen, we can infer that sulfur autotrophic denitrification bacteria play a dominant role in both reactors, and the denitrification ability of Group A is stronger than that of Group B.

### Succession of microbial community

High throughput sequencing yielded a total of 9,644,362 qualified 16S rRNA gene reads from all samples. Each sample contained a range of 24,924–51,755 clean bacterial reads (Supplementary Table 2). A total of 27,308 bacterial OTUs were clustered using a 97% identity cut-off for the target gene sequences. Subsampling was performed on a total of 24,000 reads for each sample during the OTU clustering process. The Shannon diversity index and Species richness index were used to represent the alpha diversity of the microbial consortia.

**Figure 2:**
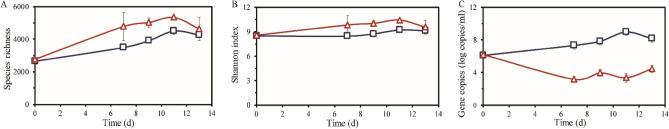
α-diversity and biomass of microbial communities within Groups A and B. The plot illustrates the changes in the species richness (**A**) and Shannon index (**B**) of bacterial communities and the abundance of bacterial biomass expressed as log(10) of rRNA gene copy numbers (**C**) during domestication.

During the domestication process, the diversity and richness of the microbial community displayed an upregulated trend in the initial 11 d, followed by a slight decrease on day 13. Notably, these indices exhibited fluctuating changes throughout the domestication period. Specifically, on day 13, the Shannon diversity index and Species richness of Group B bacteria were significantly higher than Group A (Fig. 2A and B). This difference can be attributed to the insufficient system homogeneity in Group B. The dynamic characteristics of microbial community assembly in both Group A and Group B were further explored using qPCR and amplicon sequencing analysis (Fig. 2C). The cell numbers of total bacteria in Group A exhibited an increase to 8.9 ± 0.4 log 16S rRNA gene copies/ml, followed by a decrease to 8.2 ± 0.4 log copies/ml on day 13. In contrast, for Group B, the biomass in the samples decreased to 3.2 ± 0.3 log 16S rRNA gene copies/ml on day 7. Subsequently, there was a slight fluctuation, with a value of 4.4 ± 0.5 log copies/ml reached on the 13th day. Due to differences in substrate transfer efficiency within the system, the biomass in Group A samples was approximately 4 orders higher than in Group B (Fig. 2C).

**Figure 3:**
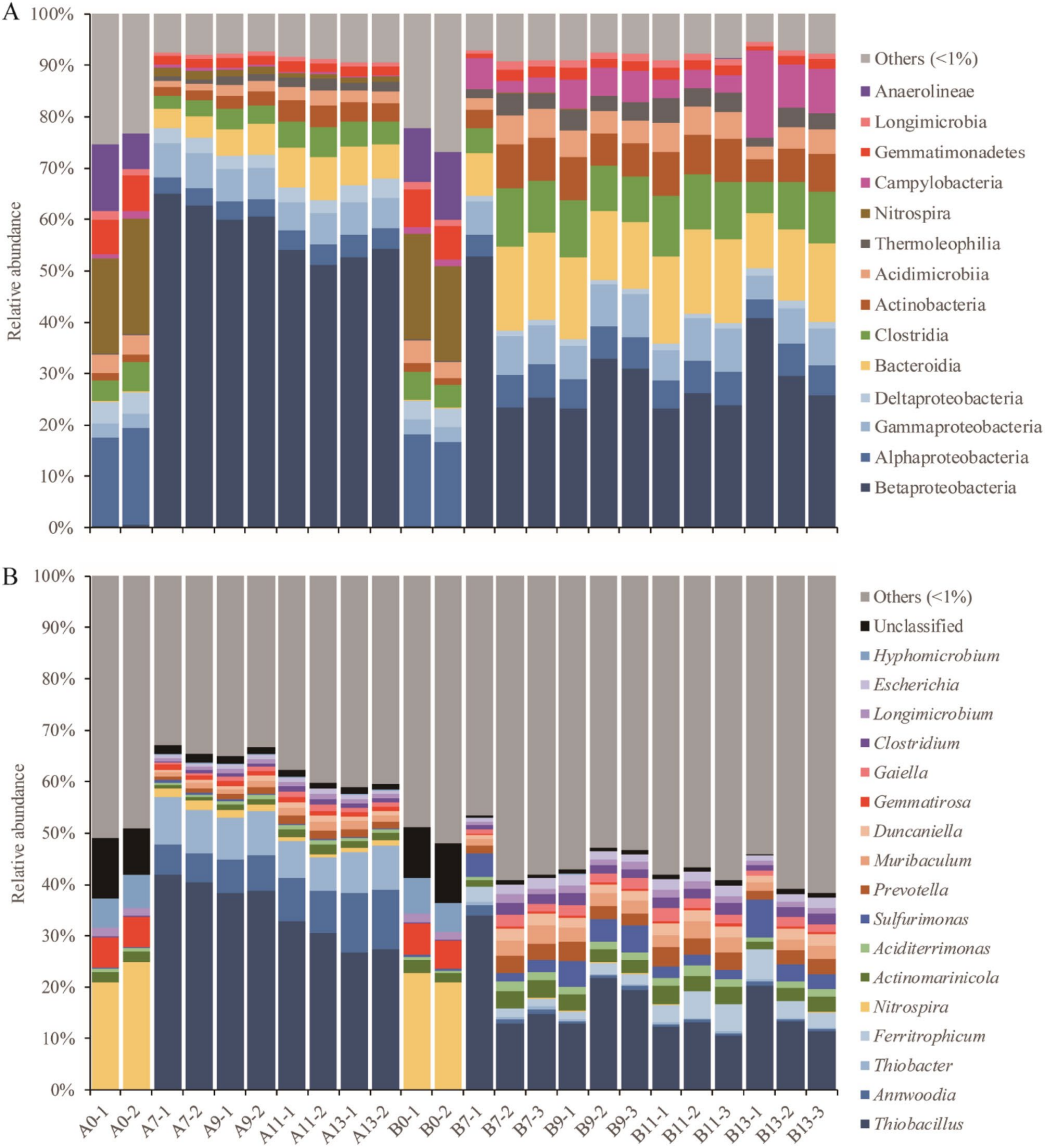
A comparison of the composition of bacterial communities in sequential samples. Library composition based on bacterial rRNA gene sequences from the Silva database. Taxonomy is presented at the class (**A**) and genus (**B**) levels. Taxon whose relative abundance is less than 1% across all samples are Grouped under others.

Figure 3 illustrates the temporal patterns of bacterial community structure during the domestication process of Groups A and B. The predominant bacterial class observed across samples was Betaproteobacteria, with an average relative abundance of 34.16%. Alphaproteobacteria accounted for 7.07% of the bacterial composition. It is noteworthy that the bacterial composition of the initial samples differed significantly from that of the samples collected during the later stages of fermentation.

In the initial samples, the dominant bacterial Groups were Nitrospira, Alphaproteobacteria, and Anaerolineae, with relative abundances of 20.13%, 17.63%, and 10.58%, respectively. During domestication, these Groups ceased to be the dominant ones. The relative abundances of Nitrospira and Alphaproteobacteria decreased to 0.54% and 4.96%, respectively, and Anaerolineae disappeared completely. On the other hand, the relative abundance of Betaproteobacteria increased from 0.37% at the beginning to 40.92%, indicating its dominance in the domesticated samples. Bacteroidia also increased relative abundance from 0.25 to 14.49% in Group B samples (Fig. 3A).

On day 0, the top three predominant genera were Nitrospira, Gemmatirosa, and Hyphomicrobium. However, their abundance decreased during the domestication process. *Nitrospira* decreased from 20.13 to 0.45% after 13 days (Fig. 3B). *Gemmatirosa* and *Hypomicrobium* also showed a decrease in abundance from about 5% to below 1%. The dominant genera in Group A were *Thiobacillus, Annwoodia,* and *Thiobacter*, with average abundances of 34.15%, 8.01%, and 7.94%, respectively. The abundances of *Thiobacillus* and *Thiobacter* peaked within 7 d and then gradually decreased. In Group B samples, the average relative abundances of *Annwoodia* and *Thiobacter* were relatively low, less than 1%. *Sulfurimonas* and *Ferritropicum* had replaced their dominant niche. The abundance of *Sulfurimonas* increased by about 10 times compared to the initial sample. *Ferritropicum* was not detected in the initial sample, but its abundance increased to 4.61% after 11 days in Group B. The findings indicate that significant changes were made to the composition of the bacterial community during the domestication process, with distinct changes in the relative abundance of different genera.

### Profiles of microbial communities between two Groups

**Figure 4:**
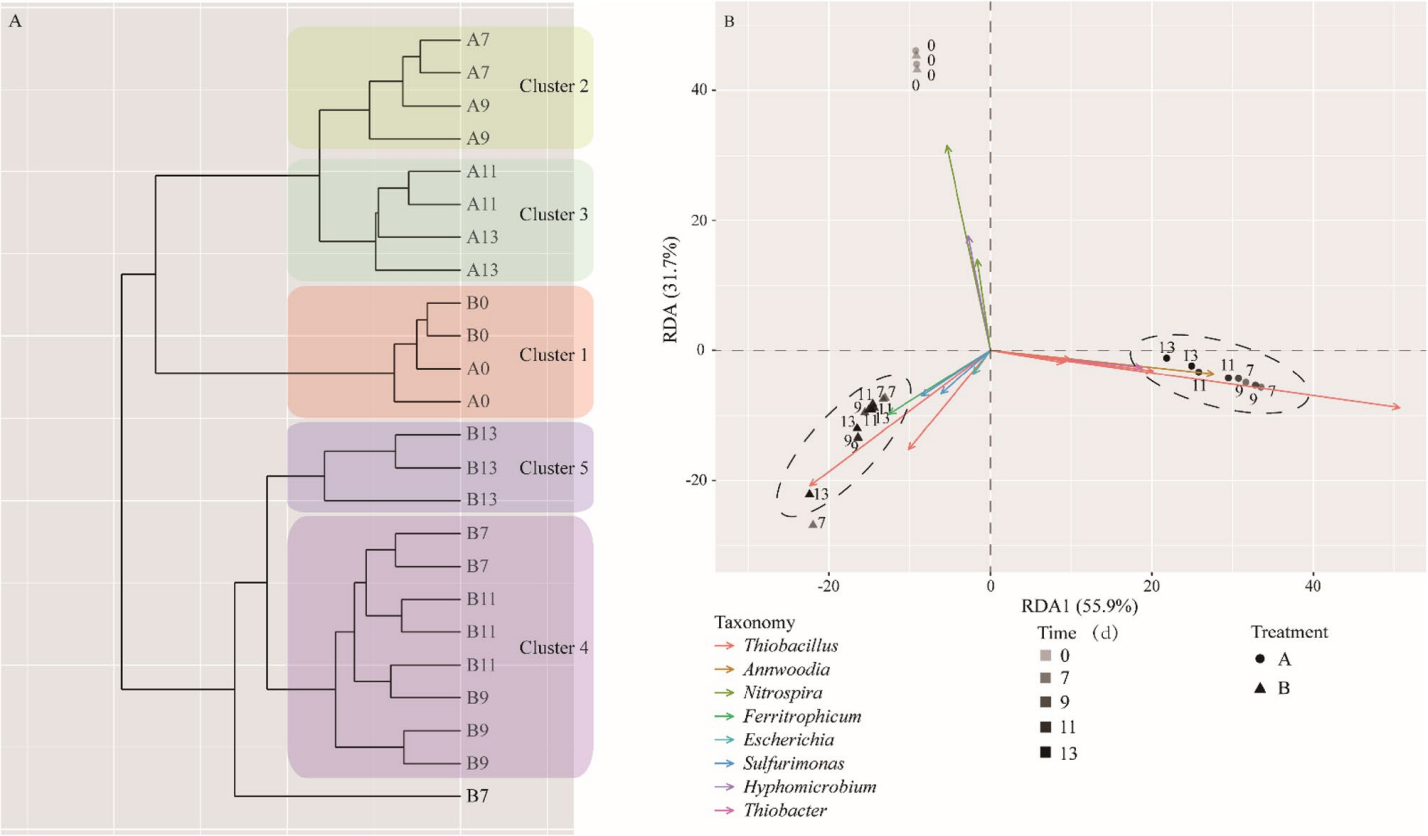
β-diversity of bacterial communities within sequential samples. Cluster analysis (**A**) of bacterial communities based on Euclidean distance algorithms performed by unweighted pair Group method (UPGMA). A redundancy analysis (**B**) of bacterial communities generated by the Euclidean distance matrix. The circles and triangles represent the community composition data for Groups A and B, respectively.

Cluster and RDA analyses were performed using the Euclidean distance algorithm to assess the dissimilarities of microbial communities between Group A and Group B during domestication. The results revealed that the profiles of sulfur autotrophic denitrification bacterial communities could be categorized into two Groups (Fig. 4A). Furthermore, the Group I could be further divided into three clusters: clusters 1 (day 0), cluster 2 (day 7–9) and cluster 3 (day 11–13). In Group II, the samples from day 7 to day 11 clustered together and were distinguishable from the samples of day 13 (cluster 5) (Fig. 4B). The RDA results analyses indicated that the microbial community dissimilarities across all samples could be explained by 87%, with the first axis (RDA1) contributing 55.1% of the discrepancy. After domestication, the RDA1 scores of the samples from Group A and Group B exhibited a significant difference. This difference was also observed in RDA2 scores (S. Fig. 1A and B). Statistical analysis using ANOSIM (analysis of similarities) supported the significant difference (P < 0.01) between Group A and Group B based on the RDA 1 score (S. Fig. 1C).

In the sorting chart, the arrows represent the influence of the top 15 OTUs on the samples. One of the top influential OTUs, OTU5, belongs to the genus *Nitrospira* and showed the highest correlation with cluster 1. Furthermore, 6 OTUs belonging to the genus *Thiobacillus* exhibited a high correlation with the domesticated samples of both Group A and Group B. It is worth noting that a specific OTU, *Thiobacter* (OTU33165), was associated with samples from Group A, while another OTU, *Ferritropicum* (OTU28108), was matched with samples and Group B. Thus, during the process of domestication, *Thiobacter* and *Ferritropicum* had a distinct presence and influence in their respective Groups.

### Indicator species analysis between two Groups

**Figure 5:**
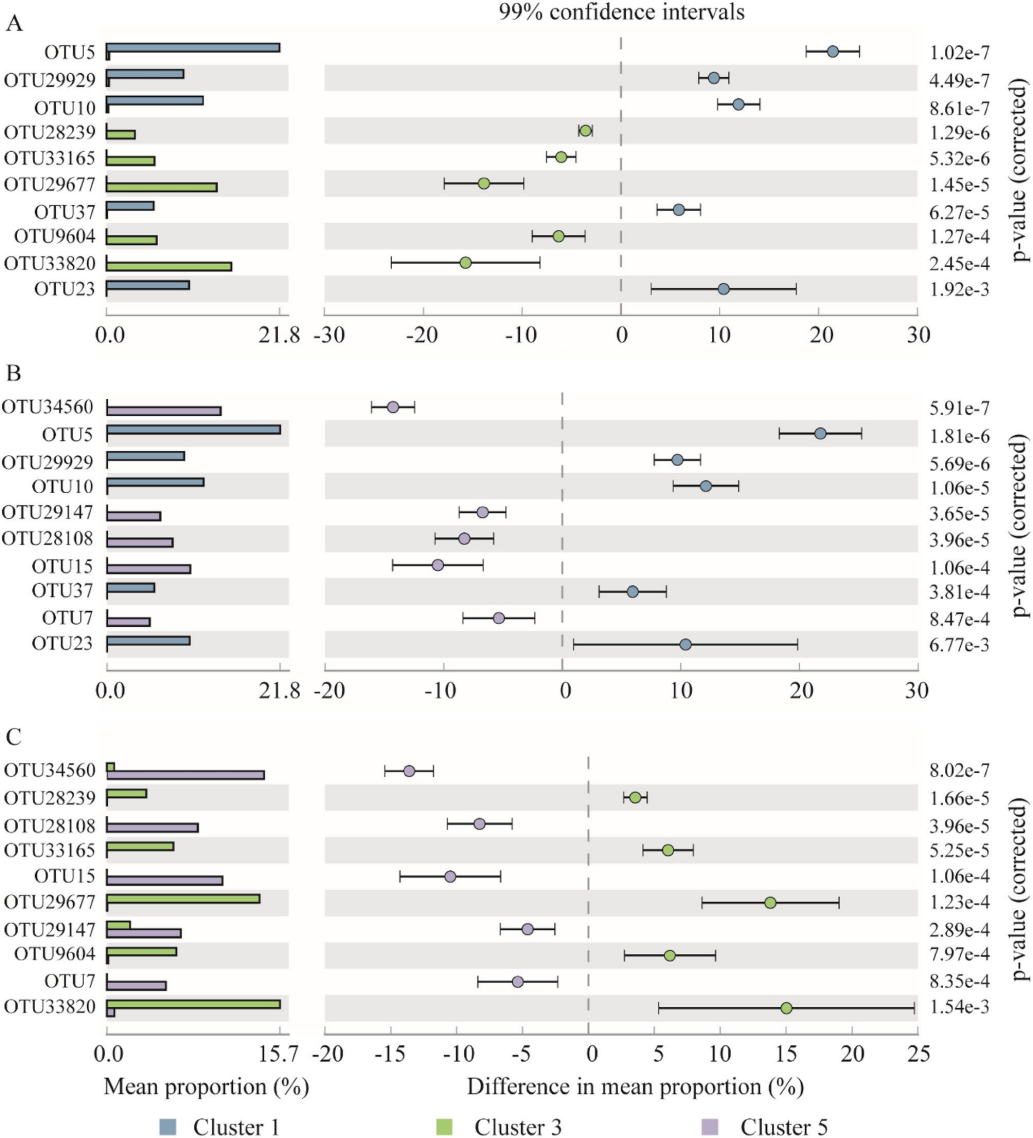
A pairwise comparison of the top ten OTUs in terms of abundance. (**A**) Cluster1 versus cluster3, (**B**) cluster1 versus cluster5 and (**C**) cluster3 versus cluster5.

We analysed using the STAMP software to explore the impact of different sulfur sources on the sulfur autotrophic bacterial community at the species level. We focused on comparing the species composition of the inoculants (cluster 1) and the later-stage samples from both treatment Groups (cluster 3 and cluster 5) based on 97% cut-off OTU. The Grouping of samples was based on the β-diversity of bacterial communities in sequential samples. Both Group A and Group B used activated sludge from the aeration tank as inoculants. Figure 5 illustrates these species-level variations and explains how different sulfur sources influence the composition of bacterial communities.

In cluster 1, the abundances of OTU5, OTU10, OTU23, OTU29929, and OTU37 were significantly higher compared to cluster 3 (Fig. 5A) and cluster 5 (Fig. 5B). These OTUs accounted for more than 5% of the initial samples but were nearly absent in cluster 3 and cluster 5. Specifically, OTU5 and OTU29929, both affiliated with *Nitrospira,* had abundances of 21.78 and 9.72, respectively, indicating their dominant presence in the community. The remaining three OTUs belonged to *Hyphomicrobium*, *Ornatilina*, and *Pseudoramibacter*, with relative abundances of 12.15%, 10.40%, and 5.94%, respectively.Three of the top 5 enriched OTUs in cluster 3 belonged to the *Thiobacillus* genus: OTU28239, OTU9604, and OTU33820. Together they accounted for a total abundance of 25.6%. The sequences of HG380547, detected in a heterotrophic and autotrophic denitrification system by Xu, was identical to that OTU28239^11^. It can be inferred that OTU28239 may have good adaptability to wastewater with different C/N ratio. However, in cluster 5, the abundance of these three OTUs was only 1/30th of that in cluster 3 (Fig. 5C). Another OTU, OTU33165, belonging to the *Thiobacter* genus, which had an abundance of 6.04% in cluster 3, was not detected in either cluster 5 or cluster 1. OTU29677, representative of *Annwoodia*, had an abundance of 13.86% in cluster 3 but was rare in cluster 5, with an abundance of less than 0.05%. As reported, *Thiobacillus* and *Annwoodia* exhibit sulfur oxidation capabilities, which suggests that they may have been involved in sulfur metabolism during domestication.

In cluster 5, two of the five enriched OTUs belonged to the *Thiobacillus* genus: OTU34560 and OTU29147. Together they accounted for a total abundance of 20.99%, 7 times more than that in cluster 3. Two OTUs belonging to the *Sulfuricurum* genus, namely OTU15 and OTU7, accounted for a total abundance of 15.85%. This abundance was 7600 and 900 times higher than in clusters 3 and 1. Furthermore, OTU28108, which belonged to *Ferritropicum*, had an abundance of 8.26% in cluster 5 and was not detected in cluster 3 or Cluster 1.

### Functional composition analysis of different clusters

**Figure 6:**
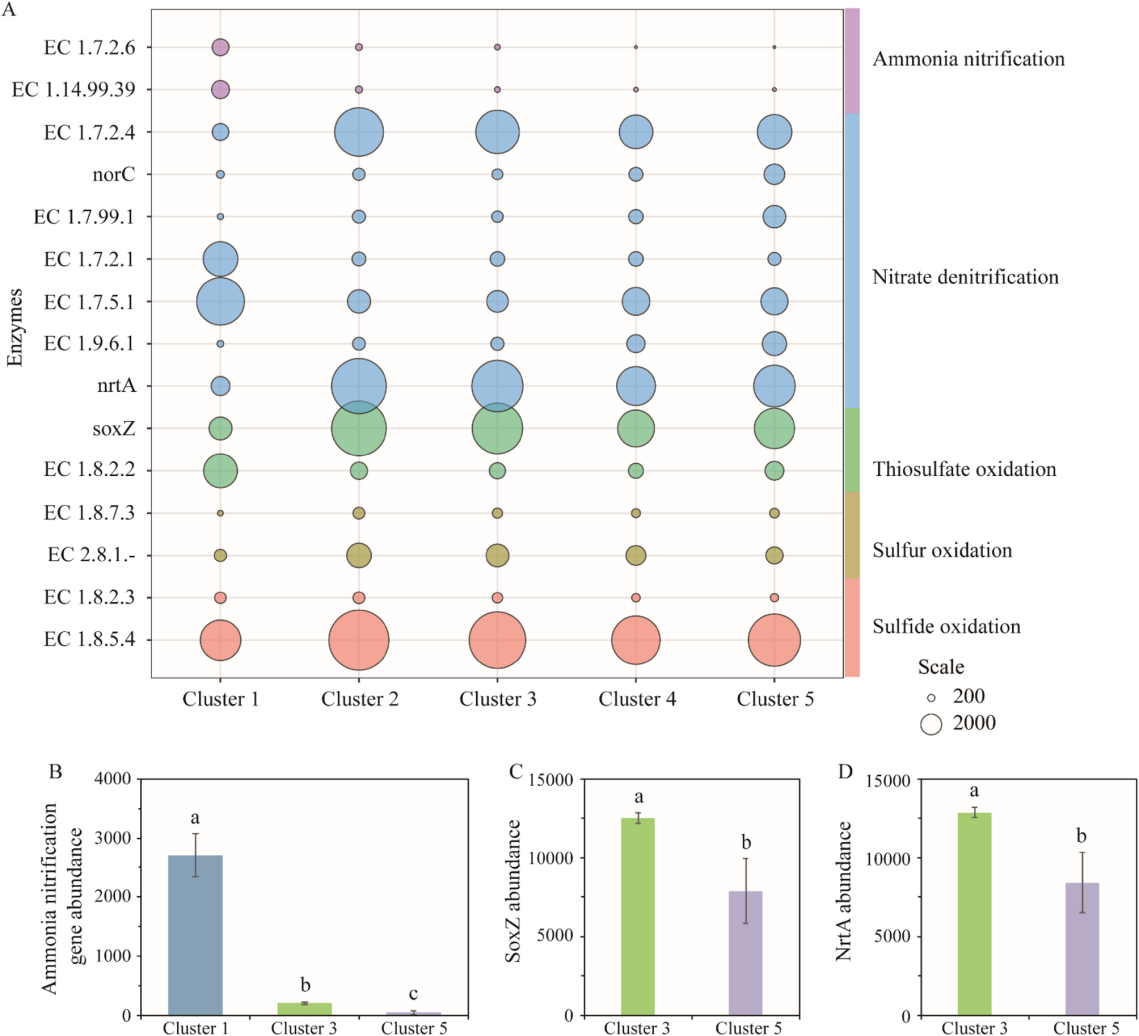
Predicted functional composition of different clusters throughout domestication, specifically focusing on nitrogen and sulfur metabolism. (**A**) Temporal patterns of enzyme read for nitrogen and sulfur metabolism in the microbial community of different clusters. The diameter of the bubble represents the read number of the enzyme. (**B**)–(**D**) One-way ANOVA of Ammonia nitrification gene reads soxZ and nrtA reads between clusters, respectively. EC 1.8.5.4, sulfide: quinone oxidoreductase; EC 1.8.2.3, flavocytochrome c sulfide dehydrogenase; EC 2.8.1.-, thiosulfate: glutathione sulfurtransferase; EC 1.8.7.3, heterodisulfide reductase; EC 1.8.2.2, thiosulfate dehydrogenase; soxZ, sulfur-oxidizing protein Z; nrtA, nitrate/nitrite transport system substrate-binding protein; EC 1.9.6.1, nitrate reductase (cytochrome); EC 1.7.5.1, nitrite oxidoreductase; EC 1.7.2.1, nitrite reductase; EC 1.7.99.1, hydroxylamine reductase; norC, nitric oxide reductase; EC 1.7.2.4, nitrous-oxide reductase; EC 1.14.99.39, ammonia monooxygenase; and EC 1.7.2.6, hydroxylamine dehydrogenase.

During the domestication process, we utilized PICRUSt2 analysis to predict the temporal functional profiles of bacterial communities based on 16S rRNA gene sequencing data. Our focus was on fifteen key enzymes involved in nitrogen and sulfur metabolism, including sulfide oxidation, sulfur oxidation, thiosulfate oxidation, nitrate denitrification, and ammonia nitrification (Fig. 6). The results demonstrated statistically significant differences (ANOSIM, P < 0.001) in the cluster-predicted metagenomes throughout the entire domestication process.

In cluster 1 of the bacterial consortia, enzymes involved in the oxidation of ammonia, such as EC 1.14.99.39 (ammonia monooxygenase) and EC 1.7.2.6 (hydroxylamine dehydrogenase), were significantly enriched (P < 0.05). This suggests that the initially inoculated bacteria primarily utilized ammonia oxidation as an energy source (Fig. 6A and B). On the other hand, in cluster 3, bacterial consortia displayed greater potential for absorbing extracellular nitrate into cells through nrtA-encoded nitrate/nitrite transport system substrate-binding protein. The intermediate product (NO_2_) was converted into N_2_ and excreted extracellularly using EC 1.7.2.4 (nitrous-oxide reductase). The abundance of this enzyme in cluster 3 was also higher than in cluster 5. These differences in enzyme abundance between cluster 3 and cluster 5 may explain the stronger denitrification capacity observed in cluster 3 during domestication (Fig. 6D).

Regarding sulfur cycling, cluster 1 exhibited a weaker ability to utilize the three forms of sulfur compared to the domesticated microbial community. Both cluster 3 and cluster 5 showed effective utilization of sulfides. Cluster 3 demonstrated a significantly stronger capacity to utilize thiosulfate compared to cluster 5 (Fig. 6C). It is noteworthy that cluster 3 also exhibited a superior ability to utilize elemental sulfur compared to cluster 5, although the difference was not statistically significant. Thiosulfate is incompletely oxidised without the SoxCD multi-enzyme complex, producing elemental sulfur^12, 13^. This might explain why the microbial communities domesticated with thiosulfate retained their ability to oxidize elemental sulfur.

## Discussion

Under similar environmental and operating conditions, this study utilized two-electron donors, sodium thiosulfate and elemental sulfur. Both systems exhibited stable performance during the 13d domestication process. However, the sodium thiosulfate system's denitrification efficiency was superior to that of the elemental sulfur system. *Thiobacillus* was the dominant bacterial Group involved in sulfur autotrophic denitrification in both systems, consistent with previous research findings.

*Anwoodia* and *Thiobacter,* enriched in Group B during the domestication process, have received limited attention in previous studies. *Anwoodia* is a newly identified genus that separated from *Thiobacillus* in 2017, *with Anwoodia aquaesulis* being the model organism^14^. It can grow heterotrophically or mixotrophically, unlike *Thiobacillus,* an obligate autotroph. Furthermore, *Annwoodia* does not produce tetrathionate during autotrophic growth during thiosulfate oxidation. In the context of combined denitrification and anaerobic ammonia oxidation processes, *Annwoodia* plays a crucial role in acquiring nitrite and contributes to a more balanced microbial community, thereby supporting effective nitrogen removal^15^. *Thiobacter,* the model organism reported in 2005, is a novel obligately chemolithoautotrophic bacterium capable of oxidizing sulfur and thiosulfate. Unlike most denitrification microorganisms that prefer weakly alkaline environments, *Thiobacter* can grow in a pH range of 5.2–7.7^16^. *Sulfurimonas,* enriched in Group B, are commonly found in habitats such as deep-sea hydrothermal vents and marine environments with high sulfide concentrations. *Sulfurimonas* species are versatile in their ability to utilize different electron donors, which allows them to play a significant role in chemical autotrophic processes and exhibit a wide distribution^17^. Through comparative genomics, Lahme et al.^18^ found that certain *Sulfurimonas* species possess only the soxCD gene clusters, enabling them to oxidize elemental sulfur to thiosulfate and sulfate.

Furthermore, when analyzing the bacterial communities at the 97% cut-off OTU level, it was observed that *Thiobacillus* exhibited high species diversity in both systems, with over 100 OTUs identified. These OTUs are all associated with sulfur autotrophic denitrification functions. Such high intrageneric species diversity within *Thiobacillus* may be attributed to the community’s functional redundancy strategy, allowing them to adapt to environmental changes effectively^19^.

Interestingly, when comparing the top 10 Thiobacillus OTUs based on their average abundance in Groups A and B, only 2 OTUs were found to overlap. The highest abundance OTU, OTU34560, in Group B, was ranked 15th in Group A, while the highest abundance OTU, OTU33820, in Group A, was ranked fifth. The significant difference in abundance between these OTUs raises questions about whether this variation is due to stochastic processes during community formation or differences in ecological niches among different OTUs^20, 21^. Further investigation using a combination of culturomics and comparative genomics may be necessary to gain a deeper understanding of these distinct OTUs. Consistent with these findings, the β-diversity analysis at the OTUs level also revealed significant differences in the bacterial community structure between Groups A and B, contradicting the conclusions drawn by Zhou and Lv^22, 23^.

The functional prediction analysis based on available genome information indicated that the microbial community in Group A exhibited stronger denitrification and sulfate utilization capabilities than Group B. Additionally, it was observed that Group A microorganisms had a slightly higher potential for utilizing elemental sulfur than Group B, likely due to the partial conversion of sodium thiosulfate into sulfur for oxdiation during the utilization process^24^. These findings were supported by experimental results from Zhou et al.^23^, who conducted studies replacing sulfur sources. Considering that the sodium thiosulfate Group promoted biomass growth, initiating the denitrification system with sulfur as the electron donor using sodium thiosulfate at the beginning could be beneficial.

This study compares the effects of sulfur sources on the community and function of sulfur autotrophic denitrifying bacteria from the perspective of species level and functional genes. The results of this study provide a sufficient experimental basis for improving the start-up time and operating cost of sulfur autotrophic denitrification system through sulfur source switching^25^. At the same time, the reasons for the differences in species of Thiobacillus in the two systems deserve further analysis and verification. Furthermore, the sulfur autotrophic denitrification system is a controllable microbial community for the investigation of microbial community succession, evolution, and horizontal gene transfer^26^.

In conclusion, despite their distinct species composition, the autotrophic denitrification bacteria utilizing sodium thiosulfate and elemental sulfur as sulfur sources exhibit strong substrate adaptability and functional consistency. This discovery offers valuable technical support for applying autotrophic denitrification using different sulfur sources and opens up new avenues for in-depth mechanistic analysis.

## Materials and methods

### Microbial cultivation and sample collection

This study utilized the initial inoculum of the seed sludge obtained from urban sewage treatment plants. Two different sulfur sources, sodium thiosulfate (Group A) and elemental sulfur (Group B), were employed as electron donors in the denitrification process.

In the experiment of Group A, a 5 l fermentation tank was utilized. The tank initially contained 150 ml of seed sludge with a volatile suspended solids (VSS) concentration of 5416 mg/l and 2 l of simulated wastewater (Supplementary Table 1). The fermentation system was maintained at 30 °C while stirring at 150 rpm. Daily, we collected 20 ml of sample from the fermentation broth, centrifuged at 5000 × g for 15 min, and filtered the resulting supernatant using a 0.22 µm filter membrane. The precipitate obtained from the centrifugation process was used for DNA extraction, which was subsequently subjected to high-throughput sequencing to analyze the microbial community structure. On the other hand, the filtrate obtained after filtering the fermentation broth was utilized to measure various physical and chemical indicators. Throughout the fermentation process, half of the simulated wastewater was replaced every day.

For Group B, we employed an upflow packed bed reactor made from a plexiglass tube with an approximate inner diameter of 10 cm and a height of around 100 cm. The reactor was filled with elemental sulfur particles, each with a diameter of about 2 mm, reaching a height of approximately 40 cm. A total of 2.0 l of simulated wastewater (without Na_2_S_2_O_3_) was continuously injected into the system from the bottom using a peristaltic pump, maintaining a constant flow rate of 1.0 l/days. The reactor was kept in a constant temperature incubator set at 30 ℃ throughout the acclimation process. Every day, about 10 sulfur particles were collected from the middle portion of the reactor. These particles were then immersed in 20 ml of fermentation broth and subjected to rotational shaking at 100 rpm for 1 h at room temperature. Afterwards, the suspension was collected by centrifugation using the same parameters mentioned earlier. The resulting precipitate obtained from this centrifugation step was utilized for further analysis^27^.

### Determination of physicochemical parameters

To analyze the pre-treated water samples, several parameters were determined. The pH of the samples was measured using a Mettler Toledo FiveEasy Plus™ pH/mV meter equipped with a LE438 solid electrode (Shanghai, China). Total nitrogen content (TN), nitrate nitrogen content (NO_3_^−^-N), nitrite nitrogen content (NO_2_^−^-N), and ammonia nitrogen content (NH_4_^+^-N) were quantified using potassium persulfate digestion combined with UV spectrophotometry, UV spectrophotometry, *N*-(1-naphthyl)-ethylenediamine spectrophotometry and Nessler's reagent spectrophotometry, respectively^28^.

### DNA extraction

The collected supernatant underwent DNA extraction using a PowerSoil® DNA Isolation Kit (Mo Bio Laboratories, USA) following the manufacturer’s instructions^29^. The quality and quantity of the extracted DNA were assessed using a NanoDrop 2000 UV spectrophotometer (Thermo Scientific, USA). Subsequently, all DNA samples were stored at – 80 °C for further analysis.

### Quantitative real-time PCR (qPCR)

Bacterial cell numbers in all samples were determined using qPCR on a CFX Connect™ Real-Time PCR Detection System (Bio-Rad, USA). For the analysis, primers Eub338 (5’-ACTCCTACGGGAGGCAGCAG-3’) and Eub518 (5’-ATTACCGCGGCTGCTGG-3’) were used to target the bacterial cell numbers^30^. The qPCR reactions were conducted using the SYBR™ Select Master Mix (Applied Biosystems, USA) following our previous protocol^31^. To establish standard curves, the Cq value was plotted against the concentration of Escherichia coli’s 16S rRNA gene, with a tenfold serial dilution for the enumeration of bacteria (Supplementary Fig. 1). The copy numbers of the 16S rRNA gene were calculated using the previously reported method^32^.

### Amplicon and Illumina MiSeq sequencing

To generate bacterial amplicon libraries, the V3-V4 hypervariable region of the 16S rRNA gene was amplified using the universal primer set 338F (5′-ACTCCTACGGGAGGCAGCA-3′) and 806R (5′-GGACTACHVGGGTWTCTAAT-3′)^33^. Following the PCR amplification, purification of the PCR products was carried out using a SanPrep Column PCR Product Purification Kit (Sangon Biotech, Shanghai, China). The purified amplicons were then used for library construction with equimolar content. Library construction was performed using a TruSeq® DNA PCR-Free Sample Preparation Kit (Illumina, USA). The resulting libraries were subjected to paired-end (2 × 300 bp) sequencing on the Illumina MiSeq platform.

The amplicon sequencing data were analyzed using the QIIME (V1.9.1) pipeline, which involved quality control, de-multiplexing, and subsequent analysis^34^. High-quality data from all samples were classified into operational taxonomic units (OTUs) using USEARCH with a 97% identity cut-off after subsampling to the same sequencing depth^35^. The representative sequences of each OTU were annotated using the SILVA database (http://www.arb-silva.de/) and RDP classifier at a confidence level of 80%^36^. Calculation of alpha diversity indices was performed using QIIME (V1.9.1). Cluster analysis, redundancy analysis (RDA), and analysis of similarities (ANOSIM) were conducted in R (version 4.2.1) using the vegan package (version 2.6-4). The functional composition of bacteria in Groups A and B during domestication was predicted using PICRUSt2^37^.

### Supplementary Information

Supplementary Information.

## Data Availability

The amplicon sequencing data sets have been deposited in the NCBI Sequence Read Archive with the accession number PRJNA967745. The data can be accessed through the following link: SRA, http://www.ncbi.nlm.nih.gov/Traces/sra.
