# Supplementary material for: Impact of different sulfur sources on the structure and function of sulfur autotrophic denitrification bacteria
Source: Sci Rep. 2023 Nov 8;13:19404. doi: 10.1038/s41598-023-46829-y (PMC10632486; doi:10.1038/s41598-023-46829-y)

Effects of Different Sulfur Sources on the Structure and Function of Sulfur Autotrophic Denitrification Bacteria

Zhenguo Chen ^1,2, †^, Minlan Lou ^1, †^, Wenting Zhu^1,3^, Jin Qiu^1^, Hongwei Chen^1^ and Wei Qian ^1,^ *

^1^ School of Chemistry and Life Sciences, Suzhou University of Science and Technology, 99 Xuefu Road, Suzhou 215009, China

^2^ Suzhou Fangzhou Environmental Protection Technology Co., Ltd, 388 Ruoshui Road, Suzhou 215125, China

^3^ State Key Laboratory of Pollution Control and Resource Reuse, School of the Environment, Suzhou Polytechnic Institute of Agriculture, 279 Xiyuan Road, Suzhou 215008, China

***** Correspondence: Qianwei@mail.usts.edu.cn

^†^ These authors contributed equally to this work.

**Supplementary Table 1.** Composition of simulated wastewater

| NaNO_3_ | 0.6071 | g/L |
| --- | --- | --- |
| NaHCO_3_ | 1.00 | g/L |
| Na_2_S_2_O_3_·5H_2_O | 1.3286 | g/L |
| NaH_2_PO_4_·2H_2_O | 0.0037 | g/L |
| Trace element solution | 1 | mL/L |

Trace element solution

| ZnSO_4_·7H_2_O | 0.43 | g/L |
| --- | --- | --- |
| CoSO_4_·7H_2_O | 0.283 | g/L |
| MnSO_4_·H_2_O | 0.845 | g/L |
| CuSO_4_·5H_2_O | 0.25 | g/L |
| Na_2_EDTA | 15.00 | g/L |
| NiCl_2_·6H_2_O | 0.19 | g/L |
| H_3_BO_4_ | 0.014 | g/L |

Adjust pH to 6.8 ± 0.1

**Supplementary Table 2.** Summary of high throughput sequencing results

| Sample name | Total raw reads | Total clean reads | Percentage (%) |
| --- | --- | --- | --- |
| A0-1 | 29,258 | 27,989 | 95.66 |
| A0-2 | 29,862 | 28,193 | 94.41 |
| A7-1 | 54,891 | 51,351 | 93.55 |
| A7-2 | 55,608 | 51,755 | 93.07 |
| A9-1 | 52,469 | 50,586 | 96.41 |
| A9-2 | 46,425 | 42,405 | 91.34 |
| A11-1 | 56,667 | 50,502 | 89.12 |
| A11-2 | 48,750 | 46,664 | 95.72 |
| A13-1 | 43,083 | 41,106 | 95.41 |
| A13-2 | 52,772 | 50,144 | 95.02 |
| B0-1 | 29,990 | 27,948 | 93.19 |
| B0-2 | 27,230 | 24,924 | 91.53 |
| B7-1 | 51,132 | 46,428 | 90.80 |
| B7-2 | 44,118 | 41,582 | 94.25 |
| B7-3 | 30,079 | 27,863 | 92.63 |
| B9-1 | 42,044 | 40,674 | 96.74 |
| B9-2 | 42,440 | 40,068 | 94.41 |
| B9-3 | 48,121 | 46,384 | 96.39 |
| B11-1 | 46,089 | 43,873 | 95.19 |
| B11-2 | 35,146 | 33,561 | 95.49 |
| B11-3 | 38,739 | 37,558 | 96.95 |
| B13-1 | 39,301 | 37,364 | 95.07 |
| B13-2 | 44,671 | 41,924 | 93.85 |
| B13-3 | 35,376 | 33,590 | 94.95 |
|  | 1024261^T^ | 964436^T^ | 94.21 ^AVG^ |

Percentage = (Total clean reads/ Total raw reads) × 100%; ^T^, total reads; ^AVG^, average value; A, group A; B, group B; numbers following A or B are fermentation days.

**Supplementary Fig. 1.** β-diversity of bacterial communities in different groups. One-way ANOVA analysis of RDA1 (A) and RDA2 (B)scores of group A and group B. Analysis of Similarities (AMOSIN) (C)of group A and group B.


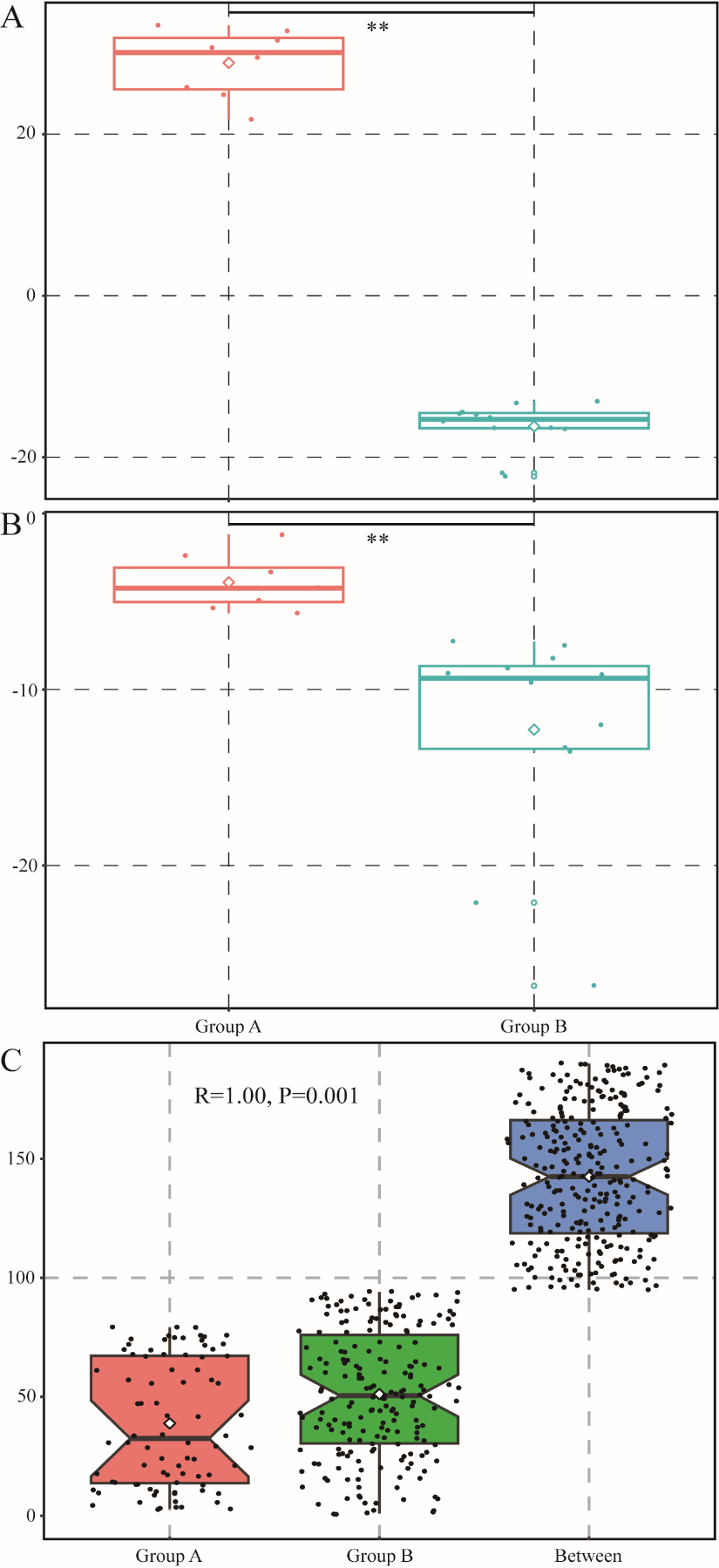

Supplement: Supplementary file 1 — Supplementary Information. [file 41598_2023_46829_MOESM1_ESM.docx]
